# Transcriptome Analysis Reveals Important Candidate Genes Related to Nutrient Reservoir, Carbohydrate Metabolism, and Defence Proteins during Grain Development of Hexaploid Bread Wheat and Its Diploid Progenitors

**DOI:** 10.3390/genes11050509

**Published:** 2020-05-05

**Authors:** Megha Kaushik, Shubham Rai, Sureshkumar Venkadesan, Subodh Kumar Sinha, Sumedha Mohan, Pranab Kumar Mandal

**Affiliations:** 1 Indian Council of Agricultural Research -National Institute on Plant Biotechnology (ICAR-NIPB), LBS Building, Pusa Campus, New Delhi-110012, India; meghakaushik3013@gmail.com (M.K.); srai2308@gmail.com (S.R.); sureshkumarv1996@gmail.com (S.V.); subsinha@gmail.com (S.K.S.); 2 Amity Institute of Biotechnology (AIB), Amity University, Sector 125, Noida, Uttar Pradesh 201313, India; smehta1@amity.edu

**Keywords:** wheat, hexaploid, diploid, transcriptome, RNA-seq, nutrient reservoir, carbohydrate metabolism, defence protein

## Abstract

Wheat grain development after anthesis is an important biological process, in which major components of seeds are synthesised, and these components are further required for germination and seed vigour. We have made a comparative RNA-Seq analysis between hexaploid wheat and its individual diploid progenitors to know the major differentially expressed genes (DEGs) involved during grain development. Two libraries from each species were generated with an average of 55.63, 55.23, 68.13, and 103.81 million reads, resulting in 79.3K, 113.7K, 90.6K, and 121.3K numbers of transcripts in AA, BB, DD, and AABBDD genome species respectively. Number of expressed genes in hexaploid wheat was not proportional to its genome size, but marginally higher than that of its diploid progenitors. However, to capture all the transcripts in hexaploid wheat, sufficiently higher number of reads was required. Functional analysis of DEGs, in all the three comparisons, showed their predominance in three major classes of genes during grain development, i.e., nutrient reservoirs, carbohydrate metabolism, and defence proteins; some of them were subsequently validated through real time quantitative Reverse Transcription Polymerase Chain Reaction (qRT-PCR). Further, developmental stage–specific gene expression showed most of the defence protein genes expressed during initial developmental stages in hexaploid contrary to the diploids at later stages. Genes related to carbohydrates anabolism expressed during early stages, whereas catabolism genes expressed at later stages in all the species. However, no trend was observed in case of different nutrient reservoirs gene expression. This data could be used to study the comparative gene expression among the three diploid species and homeologue-specific expression in hexaploid.

## 1. Introduction

The term ‘wheat’ is broadly used for diverse types of cultivated species and genotypes under the genus *Triticum* [1]. Bread wheat (*Triticum aestivum* L.), an allohexaploid species contains three different genomes (AA, BB, DD). AA (e.g., *Triticum monococcum* also known as Einkorn wheat), BB (e.g., *Aegilops speltoides*), and DD (e.g., *Aegilops tauschii*) are the three separately genome species of ancient wheat (wild relatives). Bread wheat has immense importance to humankind in the form of food livestock feed and industrial raw material [2]. With rising demand for wheat to feed the continuously growing population, global wheat security is under great strain [3]. Wheat grain development is considered as an important determinant of yield associated with flour quality. Thus, understanding the mechanism of wheat grain development to explore important candidate genes performing valuable functions during grain development are of great significance. Wild relatives of wheat could represent valuable resources to study various aspects of evolution and development that are involved in making bread wheat (as one of the most important food crops).

Grain development involves various physiological and biochemical processes that take place in different types of tissues [4]. This includes five main stages—fertilization (0 days after anthesis (DAA)), multinucleate endosperm (1–5 DAA), cellularization and early grain filling (6–13 DAA), maximum grain filling (14–28 DAA), and desiccation (25–38 DAA) [5]. Grain developmental process is very important for completing the life cycle of any crop as it accumulates different nutrient reserves in addition to embryo development and maturation. The nutritional and economic importance of wheat grain depends on the accumulation of these nutrient reserves. 

The predominant genes involved during wheat grain development are related to nutrient reserves (NRs), carbohydrate metabolism (CM), and defence proteins (DPs) [4]. NR consists of carbohydrates followed by storage proteins. However, since the metabolism of carbohydrates occurs through specific pathways involving a number of genes. CM has been designated as a separate class for studying the genes involved in anabolism and catabolism. As wheat storage proteins have highly polymorphic coding regions, wheat genotypes produce diverse types of storage proteins in terms of their quality and quantity [1]. These nutrient reserves in *Triticum aestivum* include mainly starch (60–70% of total dry grain weight (DGW)), followed by storage proteins (8–15% of DGW) [5] and lipids (2.1–3.3% of DGW) [6]. Thus, the development of wheat grain mainly depends upon the synthesis and accumulation of two major nutrients—starch and storage proteins. Storage proteins are mainly comprised of albumins, globulins, and gluten. Gluten consists of gliadins and glutenins. Further, glutenins are made of high molecular weight (HMW) and low molecular weight (LMW) proteins [1]. Carbohydrate metabolism is considered to be one of the most important metabolic processes during the wheat grain development. Studies on wheat grain proteome revealed the involvement of about 21% differentially expressed proteins (DEPs) in carbohydrate metabolism [7]. β-glucosidase is responsible for hydrolysis of carbohydrates and is considered to be one of the most abundant enzymes studied in wheat [8]. Starch, the main component of endosperm, is synthesised by coordinated reactions of four main enzymes, i.e., adenosine diphosphate ADP pyrophosphorylase, starch synthase [9], starch branching enzyme, and starch debranching enzyme [4]. 

After the accumulation of storage materials, the next important process during grain development is the protection of the developing grains. Plants may summon a variety of molecular and biochemical defence mechanisms to resist or delay many biotic and abiotic stresses [10]. Defence responses in plants are activated by incompatible interaction between host and biotic/abiotic factors that prompt several signalling pathways. Many studies have shown that amylase/trypsin inhibitors (ATIs), a class of bifunctional proteins, regulate the defence responses in wheat by blocking amylase and trypsin enzyme activities in pests [7,11,12]. The Bowman–Birk family of cysteine-rich proteases are also reportedly involved in defence responses against insect infestation [13]. 

Gene expression analysis of hexaploid bread wheat through RNA-seq and the GeneChip® Wheat Genome array revealed the expression of transcripts related to starch biosynthesis and defence proteins 2–3 weeks after anthesis (WAA); however, the most abundant transcripts from the developing seeds were related to storage proteins that continue throughout the developmental process [2,6,14].

Although the transcriptome analysis has been performed in hexaploid wheat during grain development [3,15], a detailed comparative transcriptional characterization of bread wheat and its diploid progenitors has not yet been reported. It would be highly informative to study the stage-specific expression of these important classes of genes during the grain developmental process in bread wheat and its diploid progenitors. This would help in understanding the changes that occurred in the grain quality of wheat during the evolution from ancient wheat to modern bread wheat.

In the present study, RNA-seq analyses of developing grains of bread wheat (AABBDD: PBW343) and its diploid progenitors (*T. monococcum*: AA, AN104; *Ae. speltoides*: BB, PN84; *Ae. tauschii*: DD, PN95) have been performed to examine their gene expression profiles and to monitor differentially expressed genes. Here we also report the stage specific expression of a few highly expressed candidate genes involved in the grain development of wheat belonging to three main classes, i.e., NR, CM, and DP. We have carried out these experiments to understand the molecular mechanisms involved in metabolic pathways for synthesis and regulation of crucial components of grain development that makes modern bread wheat different from its ancient diploid progenitors. Moreover, transcriptome sequencing of bread wheat as well as its progenitors offers a possible approach to study the genetics of wheat endosperm development.

## 2. Materials and Methods

### 2.1. Plant Material

Wheat seeds of different genotypes (Table 1) were procured from the Indian Institute of Wheat and Barley Research (IIWBR), Karnal, India, and the Indian Agricultural Research Institute (IARI), New Delhi, India. 

### 2.2. Growing Condition:

Healthy seeds were surface-sterilised using 0.5% HgCl_2_ for 1 min_._ After several washes with ddH_2_O, the seeds were sown in pots (18″ diameter and 24″ height) at the ICAR-NIPB net house (28^°^64″ N, 77^°^16″ E) following the standard package of practices for growing wheat. Developing grain samples were collected at 2, 3, 4, and 5 WAA. Tissues were snap frozen in liquid nitrogen and stored at -80^0^C for downstream processes. 

### 2.3. RNA Extraction and Illumina Sequencing

Total RNA from two sets of biological replicates were extracted from the frozen samples using Purelink^TM^ RNA Mini Kit (Invitrogen, Invitrogen Bioservices India Pvt. Ltd., Banglore, India). Grain samples from three different plants were pooled to form one biological replicate. For each library preparation, RNA samples from all four stages were pooled in equimolar concentration. Therefore, altogether, eight separate libraries consisting of two biological replicates of four genotypes were prepared. Illumina-compatible NEBNext® Ultra™ Directional RNA Library Prep Kit (New England BioLabs, Ipswich, MA, USA) was used for preparation of libraries. RNA-Seq was performed using Illumina (Hiseq^TM^ 2500) as per manufacturer protocol to generate 2 × 150 bp reads. The generated raw reads were submitted to the NCBI sequence read archives (SRA) bearing accession number PRJNA 470527.

### 2.4. RNA-Seq Data Processing and Differential Gene Expression Analysis

The generated raw reads were initially subjected to quality check using FastQC software (http://www.bioinformatics.bbsrc.ac.uk/projects/fastqc/) [16]. The raw reads were further processed by Cutadapt software (v.1.7.1) [17] for removal of adapters and low-quality reads (Phred Score < 30). Hisat2 (version 2.2.1.0) [18] and StringTie (version 1.3.5) [19] software were used for mapping and transcript assembly of high quality reads respectively. The trimmed reads were mapped to the latest version of the wheat reference genome (version IWGSC RefSeq v1.0, http://plants.ensembl.org/Triticum_aestivum/). The transcripts were assembled and merged together under the bearing of GFF annotation using StringTie merge function to create a common set of transcripts for all eight libraries. Further edgeR software in Blast2GO package was used for differential expression analysis with the help of assembled and merged GTF files. The differential fold change was calculated with default parameter and differentially expressed genes (DEGs) were filtered at cut off two-fold change with *p*-value ≤ 0.01.

### 2.5. Transcript Annotation, Gene Ontology and KEGG Pathway Analysis

Transcripts were annotated by the homology search against all plant protein datasets from the Uniprot database using the BLAST program with a threshold *E*-value ≤ 1.0E-3. BLASTX results were imported to Blast2GO suite for further annotation and KEGG pathway analysis. We obtained the Gene Ontology (GO) annotation of transcripts based on the non-redundant annotation and GO functional classifications for all genes using the Blast2GO package with E-value ≤ e ×10^−6^ [20]. We used the AgriGO (http://bioinfo.cau.edu.cn/agriGO/analysis.php) web-based tool for GO enrichment analysis. Each set of DEGs resulted from comparison of genotype-pair was analysed. AgriGO SEA parameter settings were as follows: fisher test, with Yekutieli (FDR under dependency), 0.05 significance level, five minimum mapping entries, and complete GO and molecular function gene ontology. 

### 2.6. Quantitative PCR Analysis

Validation of RNA-Seq data and stage specific gene expression were carried out using the qRT-PCR [21]. Primers were generated using PrimerQuest tool from Integrated DNA Technologies [22,23] (Primer sequences are provided in Tables S1 and S2) and qRT-PCR was carried out using Mastercycler® ep Realplex from Eppendorf (Hamburg, Germany). The qRT-PCR was performed for both the biological replicates with three technical replicates each. Actin was used as internal control. Since there was considerable (99%) homology in active coding region, the same primers were used for all four species.

### 2.7. Statistical Analysis

Standard error of means (SEM) was calculated and used as an error bar, wherever applicable.

## 3. Results

### 3.1. Comparative Analyses of Transcriptome Profiles during Grain Development 

To compare the changes occurring at the transcriptome level during seed development in bread wheat and its progenitors, we constructed eight RNA-Seq libraries, two from each genotype (as two biological replicates), followed by reference-based transcriptome assembly. Approximately 283 million raw reads were obtained from eight libraries ranging from 55.6 million to 103.3 million reads from each genotype (including both replications). After quality check of sequencing data and removal of adapter contaminations, a major proportion (50.81–93.19 million reads) of high-quality reads (Phred Score ≥30) were obtained, which were further used for downstream analysis. All the processed reads were aligned to the wheat reference genome and an average 60% of the reads were aligned. Transcript sequences were generated using the StringTie software. The total number of transcripts in *T. monococcum*, *Ae. speltoides*, *Ae. tauschii*, and *T. aestivum* was found to be 79,328, 113,733, 90,640, and 121,295 respectively (Table 2). Genome size of hexaploid wheat is around three times larger than that of its diploid progenitors. In the present study, the number of genes expressed during grain development was not proportional to the genome size in the hexaploid; rather, it was marginally higher than its diploid progenitors. However, we had generated almost double the number of reads in the case of hexaploid wheat than that of its diploid progenitors. To verify whether these many reads were required, we have done replication wise analysis for R1 (50M reads) and R2 (53M reads) separately in the case of hexaploid. This revealed 83,722 and 90,812 transcripts for R1 and R2, respectively, which were substantially lower than the number of transcripts obtained after merging the reads (103M).

Subgenome wise analysis of the transcripts in hexaploid wheat showed a near equal distribution among the three subgenomes with 40508 in A, 41125 in B, and 37904 in D. Further individual chromosome wise analysis of the transcripts showed that maximum number of genes was expressed in 2B and minimum in 4D chromosome (Figure S7).

### 3.2. Identification of Differentially Expressed Transcripts (DETs) during Grain Development 

To study the transcriptional variations among bread wheat and its progenitors, differential gene expression analysis was performed. A rigorous comparison at p-value ≤ 0.01, and log2 fold change ≥2 (for up-regulation), ≤ −2 (for down-regulation) was made to identify the number of DETs (Table 3) between hexaploid bread wheat and the individual diploid progenitors. The comparison was made by the expression analysis using the uniquely mapped reads.

### 3.3. Validation of Transcriptome Data

**Figure 1:**
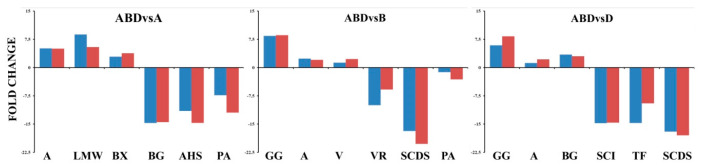
Validation of differentially expressed genes (DEGs) (blue—qPCR; red—RNAseq) in three respective comparisons (GG—gamma gliadin-B, A—avenin, V—vicilin, VR—vestitone reductase, SCDS—syn-copalyl diphosphate synthase, PA—profilin A, LMW—low molecular weight glutenin, BX—beta-D-xylosidase 4, AHS—alpha humulene synthase, SCI-—subtilisin chemotrypsin inhibitor, TF—transcription factor MyC2, BG-Beta glucosidases).

To validate the RNA-Seq results, qRT-PCR was performed for twelve key differentially expressed transcripts. Since our result showed DEGs belonging to the 3 major classes (discussed in following sections), we have randomly selected genes from each category along with a few other genes. The fold change in gene expression observed through qRT-PCR data were consistent with the changes of expression level determined by RNA-Seq (Figure 1).

### 3.4. Functional Analysis of DEGs

AgriGO uses singular enrichment analysis (SEA) for gene enrichment. SEA is the most traditional strategy for enrichment analysis using DETs. Enriched gene ontology term annotation was identified using AgriGO (http://bioinfo.cau.edu.cn/agriGO/). Statistically enriched gene ontology terms gave insights into the molecular functions that were likely to be highly active. This was derived by comparing the enriched terms to a frequency at which those GO terms appear in the transcriptome data. To classify the molecular function of DEGs, total number of up- and down-regulated transcripts between bread wheat and the individual diploid progenitors were used for GO analysis and enriched GO terms were obtained (Table 4). The background query list contained 87515 annotated genes from IWGSC (http://www.ensembl.org/). 

Further from the enriched GO terms, the most active molecular function was tabulated for better understanding (Table 5). Catalytic activity was found to have maximum DEGs in all the three comparisons (hexaploid vs. diploids). However, number of DEGs was found to be different in case of individual comparisons as mentioned below. Under the GO term ‘transcription factor; protein binding’ (GO:0000968), 61 transcripts were down-regulated only in cases of ABD *vs.* D, whereas none in case of ABD *vs.* A and ABD *vs.* B. Similarly, 30 transcripts belonging to GO term ‘nutrient reservoir’ (GO:0045735) were down-regulated in the case of ABD vs. A and none in case of ABD *vs.* B and ABD *vs.* D. A complete GO chart was generated (Figures S1–S3) with full data sets (Table S3).

**Figure 2:**
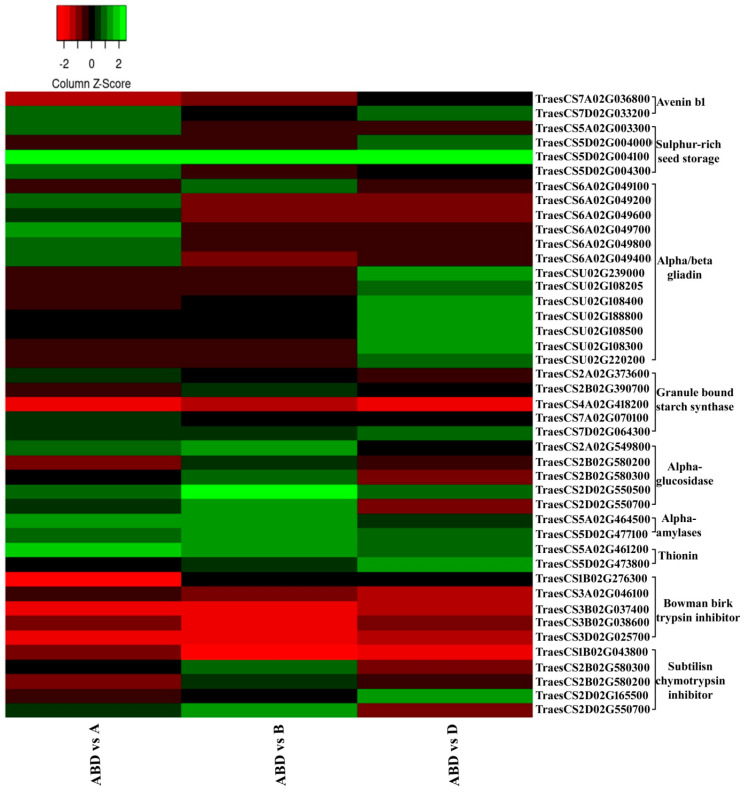
Heat map representing different transcripts having similar functions (green: up-regulated in hexaploid; red: up-regulated in diploids).

The major genes related to molecular function, which were identified through GO analysis, are of the three classes viz. nutrient reservoir (NR), carbohydrate metabolism (CM), and defence protein (DP). Our data revealed that most of the genes have their homoeologous transcripts in hexaploid wheat, and the gene have different level of expression for different homologues. Many homoeologous transcripts of hexaploid wheat showed contrasting expression even in diploid progenitors. In some instances, different transcripts belonging to same function were present on different locus, indicating multiple copies of the gene. We have constructed a heat map (Figure 2) with three classes of genes (NR, CM, DP) with examples of some of its transcripts IDs which are either homoeologous or different copies of a gene. These transcripts are differentially expressed in all the three comparisons and their fold change in each comparison was given in Table S6.

Interestingly, there were some cases like Avenin where 7A homeologue was overexpressed in *T. aestivum,* whereas 7D was overexpressed in *T. monococcum and Ae. tauschii*. Though none of the avenin transcripts could be mapped to B genome of hexaploid wheat, both the transcripts were overexpressed in *Ae. speltoides*. When these sequences were separately mapped on *Ae. speltoides* genome, we found the presence of these transcripts’ sequences in BB genotype. This was probably because of deletion of the B homologue of this gene from hexaploid wheat during evolution. 

Physiological processes work to keep a plant alive and functioning. To understand these processes during grain development, the Blast2GO package was used to identify the DEGs, which were significantly enriched in Kyoto Encyclopedia of Genes and Genomes (KEGG) pathways (Table 6). 

Most of the correlative genes were differentially expressed for starch and sucrose metabolism, phenylpropanoid biosynthesis, galactose metabolism, purine metabolism and oxidative phosphorylation. Among all the pathways, the largest pathway was phenylpropanoid synthesis followed by starch and sucrose metabolism, oxidative-phosphorylation and galactose metabolism. The number of total DEGs involved in each pathway was listed in Table 7. Total number of KEGG pathways involved in three different comparisons was generated (Figure S4-S6) with complete data sets (Table S4).

Through KEGG pathway analysis we found that most of the DEGs were mapped to starch and sucrose metabolic pathway. Transcripts of two major starch degrading enzymes i.e. α-glucosidase (TraesCS2A02G548600) and α -amylase (TraesCS5B02G475700) were up-regulated with more than 9 log_2_fold in ABD vs. A and ABD vs. B. Similarly, a starch biosynthesis enzyme i.e. granule bound starch synthase (TraesCS4A02G418200) was down-regulated with less than 9 log_2_fold in all the three comparisons.

We also found that polyphenol oxidase (TraesCS2B02G491000) and o-acyltransferase (TraesCS3A02G013600), related to defence proteins, involved in the cutin, suberine, and wax biosynthesis pathways, were up-regulated with more than 7 log_2_fold in all the three comparisons. 

### 3.5. Highly Up- and Down-Regulated Transcripts

**Figure 3:**
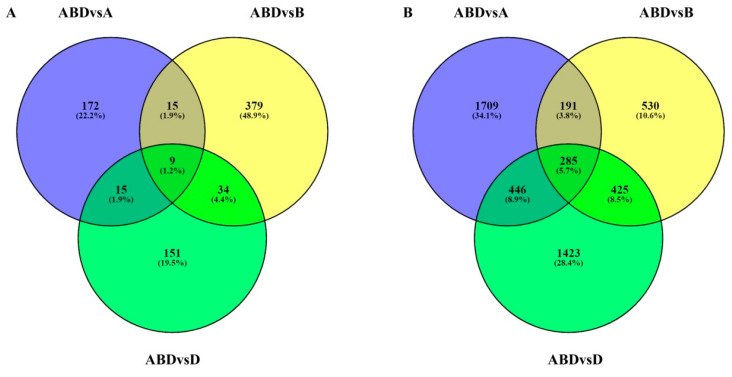
Differentially expressed transcripts (log2fold > 10) in ABD vs. A, ABD vs. B and ABD vs. D. (**A**) Nine common differentially up-regulated transcripts; (**B**) 285 common differentially down-regulated transcripts

Present comparative transcriptome analysis revealed a number of highly up- and down-regulated (log2fold>10) DEGs. These DEGs belong to different classes of protein including the above mentioned three (NR, CM, and DP) categories. However, the number of down-regulated DEGs in hexaploid was significantly higher than that of the up-regulated ones (Figure 3). This means that all the three diploid progenitors showed a number of highly up-regulated genes in comparisons to bread wheat. It was observed that there were nine up- and 285 down-regulated (common) transcripts in hexaploid wheat in comparison to its diploid progenitors. A few prominent up-regulated ones were F-box protein (defence against abiotic stresses), sulphur rich seed storage protein (nutrient reservoir), and a transcriptional co-repressor protein (represses the root promoting genes and allows panicle formation). Similarly, jacalin-related lectins (defence proteins) and syn-copalyl diphosphate synthase (enzyme involved in gibberellins synthesis) genes were highly down-regulated (~17 log_2_fold) in bread wheat in comparison to its progenitors. Detailed list of the highly up- and down-regulated transcripts are given in Table S5.

### 3.6. Stage-Specific Expression Analysis of Highly Up- and Down-Regulated Genes 

**Figure 4:**
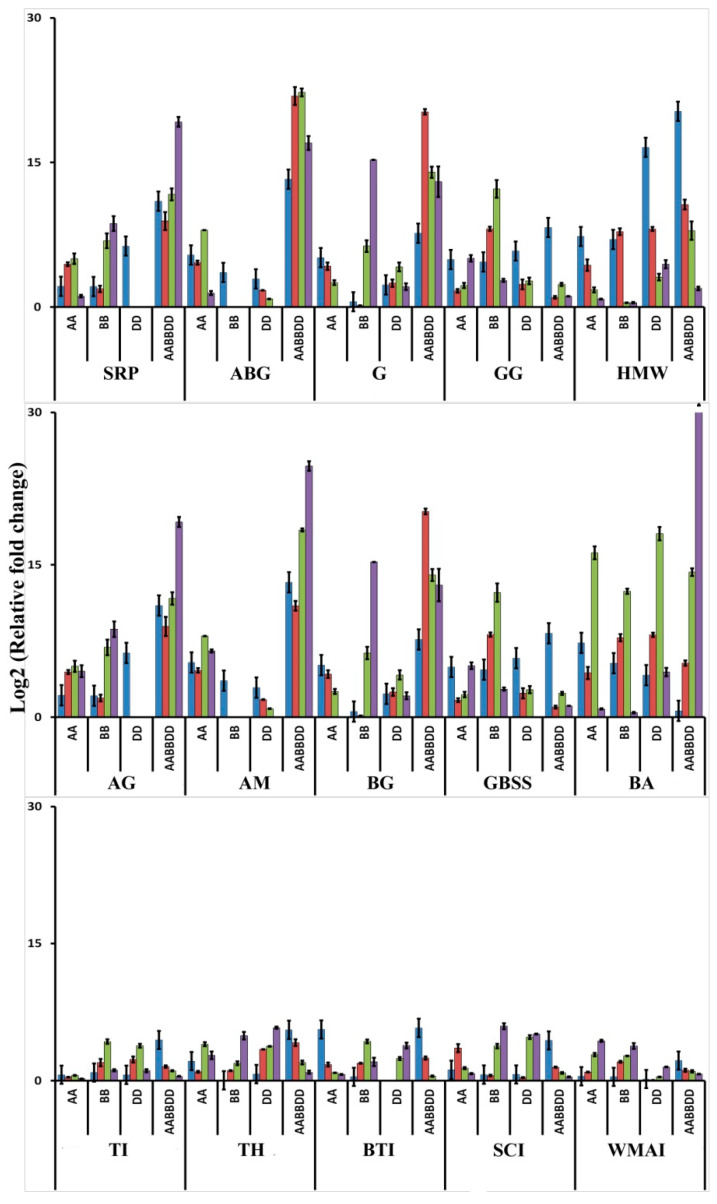
Stage-specific expression (blue—2WAA; red—3WAA; green—4WAA; purple—5WAA) of highly expressed genes belonging to three major classes (NR, CM, and DP) (SRP—sulphur rich seed storage protein; ABG—alpha/beta gliadin; G—germin; GG—gamma gliadin; HMW—high molecular weight glutenin; AG—alpha- glucosidase; AM—alpha-amylase; BG—beta-glucosidase; GBSS—granule bound starch synthase; BA—beta-amylase; TI—trypsin inhibitor CMe; TH—thionin; BTI—Bowman-Birk trypsin inhibitor; SCI—subtilisin chymotrypsin inhibitor; WMAI—wheat monomeric amylase inhibitor)

We manually screened and identified a few >8-fold DEGs belonging to three main categories (NR, CM, and DP) to study their stage specific expression during grain development (Figure 4). Almost all the genes studied were expressed at every growth stage with different trend of expression with different genes and species. Among the storage proteins, expression of glutenin genes was highest in bread wheat in all the stages; however, it showed decrease in the gene expression during grain development in all species. Contrastingly, genes encoding S-rich seed storage proteins and alpha/ beta gliadin showed a gradual increase in expression during maturity. The expression of another NR germin increased during the mid-developmental stages followed by its reduction. Carbohydrate metabolizing enzymes like α-, β-amylases and α-, β- glucosidases mainly expressed at the later stages of grain development in all the species with a few exceptions in some genotypes (e.g. *Ae. speltoides* in case of α-amylases, *Ae. tauschii* in case of α-glucosidases and *T. monococcum* in case of β- glucosidases expression). Granule bound starch synthases expressed mainly at the early stages of development except in BB, where this gene expression gradually increased till 4WAA and then reduced. Interestingly, the expression of the defence proteins in bread wheat was highest during 2WAA and gradually decreased as the plant progressed towards maturity; whereas the expression was gradually increased towards maturity in almost all the diploid progenitors. However, *T. monococcum* showed higher expression at early stages with respect to some genes (e.g., Bowman-Birk trypsin inhibitor, subtilisin inhibitor, trypsin inhibitor CMe). 

## 4. Discussion

This study was carried out mainly to understand how the expression of important genes differs during the grain development in hexaploid bread wheat when compared with its three diploid progenitors using the transcriptomics approach. Mapping of all the transcripts (reads) from all the four species were carried out against the latest version of the available genome sequence of *T. aestivum* (IWGSC RefSeqv1.0, ftp://ftp.ensemblgenomes.org/pub/plants/release-44/fasta/triticum_aestivum/dna/). This is mainly because the DEGs would be available only when the gene IDs are the same, which was possible only by mapping in the same reference sequence, rather than individual references of *T. monococcum* (AA), *Ae. speltoides* (BB) and *Ae. tauschi* (DD) and *T. aestivum* (AABBDD). Wheat grain development has five major stages, *viz*. fertilization (0 days after anthesis (DAA), multinucleate endosperm (1-5 DAA), cellularization and early grain filling (6–13 DAA), maximum grain filling (14–28 DAA), and desiccation (25–38 DAA) [3]. As the transition during grain development starts about 2 WAA [24]; samples were collected at 2 WAA, 3 WAA, 4 WAA, and 5 WAA. Primarily, we intended to study the grain development process as a whole; hence, the samples collected at different stages were pooled for RNA sequencing. From the analysed data, a few important genes were further studied for their expression at four different developmental stages using qRT-PCR. 

### 4.1. RNA-Seq Analysis of Hexaploid Wheat and Its Diploid Progenitors

#### 4.1.1. Transcriptome Data: Global View

In our experiment, we generated 55.62, 55.92, 68.13, and 103.31 million reads for AA, BB, DD, and AABBDD genome species respectively, considering the differences in genome sizes. Despite of having a big difference in the genome size (4.9–7 GB) [25,26,27,28,29,30] and the number of genes (50,000–1,00,000) [25,26,31,32] between the diploid progenitors and the hexaploid bread wheat, the total number of transcripts obtained was 79.3 K in AA, 113.7 K in BB, 90.6 K in DD, and 121.3 K in AABBDD genome species. The number of expressed genes in hexaploid wheat was not proportional to its genome size, but marginally higher than that of its diploid progenitors. It has also been reported that the genome size and the gene expression are not proportionate in the case of polyploids species [28,33,34]. However, analysis of half the number of reads in hexaploid wheat (when analysed 50 M and 53 M reads for R1 and R2 separately) revealed substantially less transcripts. Hence, it is important to generate sufficiently higher number of reads for hexaploid wheat to capture all the transcripts. 

Our analysis showed that among the three progenitors, *Ae. speltoides* (BB) expressed a higher number of transcripts (Table 1). However, when sub-genomic analysis was carried out, the number of transcripts in the B genome of hexaploid bread wheat was found to be marginally higher (Supplementary S1; figure S7).

#### 4.1.2. Functional Analysis of DEGs

The major classes of proteins whose gene expression have been analyzed in the present study include NM, DP and CM. Storage proteins which are produced mainly during grain development includes albumin, globulin, prolamin and glutenin [1,35,36]. GO analysis of our transcriptome data indicated that nutrient reservoir activity was one of the significantly enriched term in hexaploid bread wheat (*Triticum aestivum*, AABBDD) when compared to its diploid progenitors. When the ancient diploid wheat was compared with modern bread wheat, a 2S sulphur-rich seed storage protein, an albumin, was highly up-regulated in bread wheat (14, 12 and 13 log_2_fold compared to AA, BB, DD genome species respectively). Hence, among the diploids progenitors, expression of this gene was higher in *Ae. speltoides* (BB). Prolamins are proline and glutamine rich proteins which accounts for approximately half of the grain nitrogen content in wheat [25,36,37]. Gliadins are the most ancient wheat nutrient reservoir, which belongs to the prolamin family [38]. These proteins showed up-regulation in the range of 7 to 15-log_2_-fold in bread wheat (AABBDD) as compared to *T. monococcum* (AA). Transcriptome comparison of bread wheat with *Ae. speltoides* (BB) and *Ae. tauschii* (DD) showed up-regulation of 7 to 8 log_2_-fold and 3 to 14 log_2_fold for γ-gliadin B and α/β gliadin respectively. In this case, diploid progenitor AA genotype showed lower expression of gliadin genes. HMW glutenins also expressed more than 8 log_2_-fold change in bread wheat as compared to its diploid progenitors. Gliadin and glutenin together forms gluten, which is considerably higher in bread wheat as compared to its diploid progenitors. Glutenins are a potential cause for wheat intolerance [1,37] and quite evident from gene expression of HMW glutenins. Stage-specific analysis of different NR proteins did not show uniform trend of expression during grain development. However, individually each protein had a different pattern of expression from 2-5 WAA. α−β gliadins expression was corroborated with previously reported promoter, known to be active from 11 days after anthesis to 4WAA in wheat endosperm [39]. HMW glutenins also showed maximum expression during early grain filling (in all the four wheat genome species), which had also been reported in *Norin 61* bread wheat [40]. 

Wheat grain contains 65–80% carbohydrate, mostly composed of starch [1]. Accumulation of the carbohydrates takes place during the grain development. Along with carbohydrate biosynthesis, catabolism of it is equally important in the actively developing grains for supplying required energy. So, overall carbohydrate metabolism (anabolism+catabolism) is one of the major activities during wheat grain development. As anticipated, many carbohydrate metabolism related genes had been differentially expressed among bread wheat and its diploid progenitors. Starch biosynthesis has been accomplished by two classes of enzymes: granule bound starch synthase (GBSS, which is also known as ‘waxy protein’ due to the ‘waxy’ nature of amylose synthesized by this enzyme) and starch synthases (SS, involved in the synthesis of amylopectin) [41,42]. Our data showed highly down-regulation of GBSS-I (9–13 log_2_-fold) and up-regulation of SS (2–4 log_2_-fold) genes in hexaploid wheat when compared to its diploid progenitors. This result suggests relatively lesser amylose and more amylopectin synthesis in hexaploid bread wheat during grain development than that its progenitors. Results also support the fact that the bread wheat genotype PBW 343, used in the present study, is a non-waxy wheat [43]. Major starch accumulation usually occurs during the early stages of grain development in bread wheat [44]. Stage specific GBSS expression data was also correlated with this fact in *T. aestivum* (AABBDD) and *Ae. tauschii* (DD). Starch degradation in the endosperm produces glucose, which is further used by the scutellum for embryo growth [45]. A few important and abundant enzymes of starch metabolism such as α−glucosidases and AMY3 amylase [46] gene expression were more than 10 log_2_-folds lower in *T. monococcum* (AA) and *Ae. speltoides* (BB) when compared to hexaploid bread wheat. Overall stage specific analysis revealed the gene expression of carbohydrate catabolic enzymes like amylases and glucosidases were higher at later stages, whereas carbohydrate anabolic enzymes like granule bound starch synthases were higher at early stages of grain filling. This suggests that, even though carbohydrate biosynthesis generally starts at an early stage of grain filling, utilization of these carbohydrates for production of energy (ATP) starts during later stages. This is mainly because the supply of carbohydrate (in the form of sucrose) from the source gets reduced. Hence, for energy required for metabolic activities during later stage/ towards maturity has to be derived from the reserve carbohydrate available in the grain [47]. 

Wheat grain is a reservoir of mostly carbohydrates followed by proteins, which together represents more than 80% of the grain weight. There is a need to protect these nutrient reservoirs and retain them intact till germination. Defence proteins play a major role in the protection of the above nutrient reservoirs in wheat grains against biotic and abiotic factors. These defence proteins get accumulated during the grain development along with the carbohydrates and other storage proteins. When different stages of grain developments are compared in bread wheat, many DEGs related to defence proteins are reported [4,48]. However, so far, no comparative studies have been made elsewhere, between bread wheat and its different diploid progenitors during grain development. Quite a few defence related transcripts were observed in our RNA-seq data. A trypsin inhibitor, which is involved in resistance against herbivorous pests [49], was found to be up-regulated in hexaploid wheat to the extent of 14, 7, and 6 log_2_-fold when compared to *T. monococcum* (AA), *Ae. speltoides* (BB) and *Ae. tauschii* (DD) respectively. This indicated a near-equal expression in both *Aegilops* species (BB and DD), which is higher than the expression in *T. monococcum* (AA). Stage specific analysis of this gene in diploid progenitors also showed exactly similar trend. A defence protein thionin, also known to be as low molecular weight antimicrobial peptides [50,51] was found to be up-regulated more than 14 log_2_-fold in bread wheat. However, the stage specific expression revealed that the expression was more at the initial stages in hexaploid, whereas at later stages in diploids. Our data also revealed some defence proteins to be down-regulated in hexaploid wheat like Bowman Birk type trypsin inhibitors (pathogen inactivator) [52,53], subtilisin chymotrypsin inhibitors (known to inhibit insect larvae) [54] and wheat monomeric amylase inhibitors (involved in non-celiac gluten sensitivity) [55]. Stage-specific analysis of all the defence related genes revealed their gene expression in hexaploid to be higher at the initial stages and in diploids at later stages of grain filling.

The term ‘homeology’ is used to denote the relationship among corresponding subgenomes derived from different species in an allopolyploid [56]. Identifying the homeologues is a key to study the evolutionary correspondence between genes in polyploids. Our RNA-seq data could be a useful source to derive the homologue specific expression of hexaploid bread wheat during grain development, though we had not carried out the detailed analysis as our focus was mainly on comparative analysis of bread wheat and its diploid progenitors. However, expression of a few genes was compared between the homeologues from FPKM values (data not shown). Avenin b1 had two transcripts from A and D homeologues of bread wheat with ~3-fold higher expression in D than that of A genome. Similarly, X6 isoform of high molecular weight glutenin also had two transcripts from B and D homeologues and their relative expression analysis showed 4-fold over expression of B in comparison to D homeologue. 

The result of enrichment and pathway analysis of DEGs from our study may contribute towards understanding the function of genes in relation to grain development. The phenyl propanoid biosynthesis pathways were one of the top KEGG pathways identified in this comparative study. Phenyl propanoids are secondary metabolites, essential for plant survival under different biotic and abiotic stresses [57]. This pathway serves as a starting point for the production of several important compounds in plants such as flavonoids, coumarins, and lignins [58]. The second most abundant pathway was related to starch and sucrose metabolism. This pathway was also reported with ample number of DEGs involved during grain development of bread wheat [3]. The other enriched pathways from our study were oxidative phosphorylation and galactose metabolism, which are mainly involved in providing energy to the developing grains [59]. 

#### 4.1.3. Highly Up- and Down-Regulated Transcripts 

When the three sets of DEGs (between hexaploid and individual diploids) were compared, 285 common genes were down-regulated, whereas only 9 common genes were up-regulated. Among the prominent up-regulated DEGs, sulphur rich seed storage protein [59,60,61] related to NR; and F-box protein, phospholipase-D, cytb6/f complex subunit 4 [62,63,64] related to DP were most prominent. However, none of the DEGs of carbohydrate metabolism were observed to be upregulated. We also did not find any down-regulated DEGs related to nutrient reservoirs. However, Jacalin related lectins, BTIs and defensin genes [13,53,65,66,67,68] under the DP class; and glucan1,3- beta glucosidases, exopolygalatouronase [8] under CM class were found be highly down-regulated. Other than these three classes, many significant transcripts were also observed to be up- and down-regulated, which regulates the grain development process (Table S5)

## 5. Conclusions

We report, for the first time, comparative RNA-seq analysis of bread wheat and its three diploid progenitors during their grain development. This study suggests that the expression of the three major classes of genes (NR, CM and DP) dominate during grain development and number of other genes are associated and/or involved in different biological functions. Further this analysis could be useful to study the comparative gene expression among the three diploid species and also homologue specific expression in hexaploid.

## Figures and Tables

**Table 1:** Different diploids and hexaploid wheat accessions used in the study.

Genotypes	Genome	Variety Name/Accession No.
*Triticum monococcum*	AA	AN104
*Aegilops speltoides*	BB	PN84
*Aegilops tauschii*	DD	PN95
*Triticum aestivum*	AABBDD	PBW343

**Table 2:** Summary of RNA-Seq libraries generated from bread wheat (AABBDD) and their progenitors (AA, BB, DD).

Wheat Genotype	Total Raw Reads (in millions)	Processed Reads (in millions)	Alignment (%)	Expressed Transcripts
AA: *T.monococcum* (AN104&PKM1)	55.63	50.81	45.73	79328
BB: *Ae*. *speltoides* (PN84&PKM2)	55.23	49.55	54.48	113733
DD: *Ae.* *tauschii* (PN95&PKM3)	68.13	61.98	64.64	90640
AABBDD: *T. aestivum* (PBW343&PKM5)	103.31	93.19	71.44	121295

**Table 3:** Differentially expressed transcripts among bread wheat and its diploid progenitors.

Comparison	ABD *vs.* A Up Down		ABD *vs.* B Up Down		ABD *vs.* D Up Down	
Number of DETs	7993	22052	8405	16354	14657	24616

**Table 4:** Total up and down-regulated transcripts for molecular function and number of enriched GO terms.

Comparison	Molecular Function DEGs Up Down		Enriched GO Terms (Up-Regulated) at FDR<0.05	Enriched GO (Gene Ontology) Terms (Down-Regulated) at FDR<0.05
ABD *vs.* A	4549	2526	127	76
ABD *vs.* B	4099	2507	95	52
ABD *vs.* D	8296	9577	132	131

**Table 5:** Major GO term activities derived from DEGs comparing ABD *vs.* A, B, and D separately.

Enriched GO Terms Activity	ABD *vs.* A Up Down		ABD *vs.* B Up Down		ABD *vs.* D Up Down	
Nutrient reservoir (GO:0045735)	36	30	35	0	60	0
Transcription factor protein binding(GO:0000968)	50	0	27	0	63	61
Catalytic activity (GO:0003824)	2121	1228	1969	1186	3613	4563
Signal transducer (GO:0004871)	42	42	55	26	122	116
Molecular function regulator (GO:0098772)	91	56	142	56	187	230
Molecular transducer (GO:0060089)	43	41	56	27	119	118
Carbohydrate transporter (GO:1901476)	14	12	16	9	35	0

**Table 6:** Number of DEGs and pathways involved in each comparison.

	ABD *vs.* A Up Down		ABD *vs.* B Up Down		ABD *vs.* D Up Down	
Total number of DEGs	1628	2625	1441	1617	2810	3014
Total Pathways involved	209	303	167	258	398	317

**Table 7:** Clusters of KEGG (Kyoto Encyclopedia of Genes and Genomes) functional classification of DEGs.

Pathways	ABD *vs.* A	ABD *vs.* B	ABD *vs.* D
Pehnylpropanoid biosynthesis	247	208	304
Starch and sucrose metabolism	202	160	248
Oxidative Phosphorylation	139	97	166
Galactose metabolism	120	99	202
Ubiquinone and other terpenoid-quinone biosynthesis	36	34	63
Cutin, suberine, and wax biosynthesis, glycerolipid metabolism	47	31	42
Purine metabolism	28	308	677

## Supplementary Materials

The following are available online at https://www.mdpi.com/2073-4425/11/5/509/s1, Figures S1–S3: Complete GO charts of all three comparisons (up- and down-regulated), Figures S4–S6: KEGG graphs (up- and down-regulated), Figure S7: sub-genome analysis, Table S1: List of genes selected for validation and their primer sequences, Table S2: List of genes selected for stage specific expression and their primer sequences, Table S3: GO enrichment analysis, Table S4: KEGG pathway analysis, Table S5: List of common up- and down- regulated transcripts, Table S6: Fold change of different Transcript Ids.
