# Supplementary material for: Transcriptome Analysis Reveals Important Candidate Genes Related to Nutrient Reservoir, Carbohydrate Metabolism, and Defence Proteins during Grain Development of Hexaploid Bread Wheat and Its Diploid Progenitors
Source: Genes (Basel). 2020 May 5;11(5):509. doi: 10.3390/genes11050509 (PMC7290843; doi:10.3390/genes11050509)
Supplement: Supplementary file 1 [file genes-11-00509-s001.zip › Supplementary files/Figure S1-S7, Table S1, Table S2-final.docx]

Transcriptome Analysis Reveals Important Candidate Genes Related to Nutrient Reservoir, Carbohydrate Metabolism, and Defence Proteins during Grain Development of Hexaploid Bread Wheat and Its Diploid Progenitors

Megha Kaushik ^1, 2^, Shubham Rai ^1^, Sureshkumar Venkadesan ^1^, Subodh Kumar Sinha ^1^, Sumedha Mohan ^2^ and Pranab Kumar Mandal ^1,^ *

Supplementary Materials


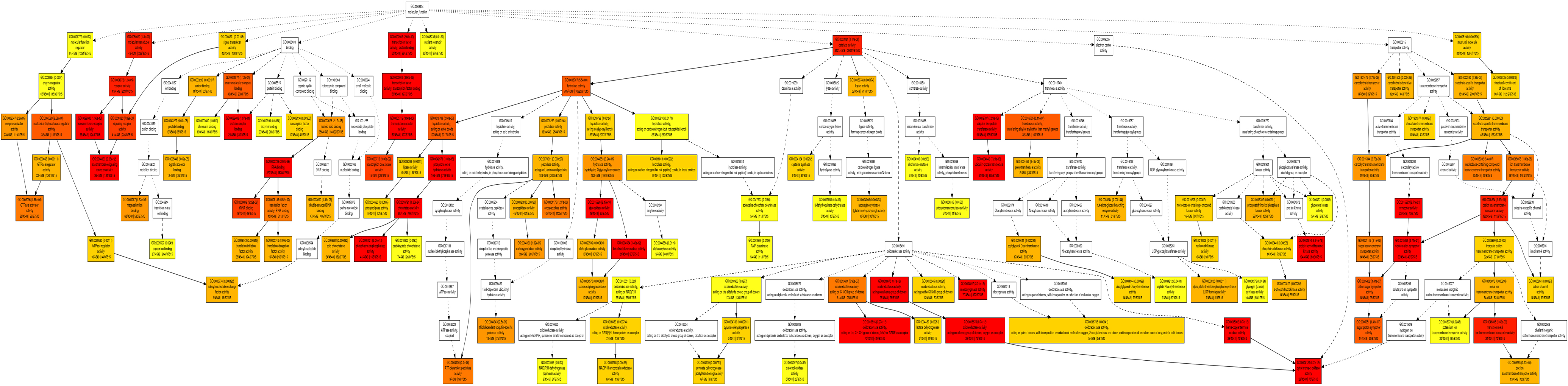

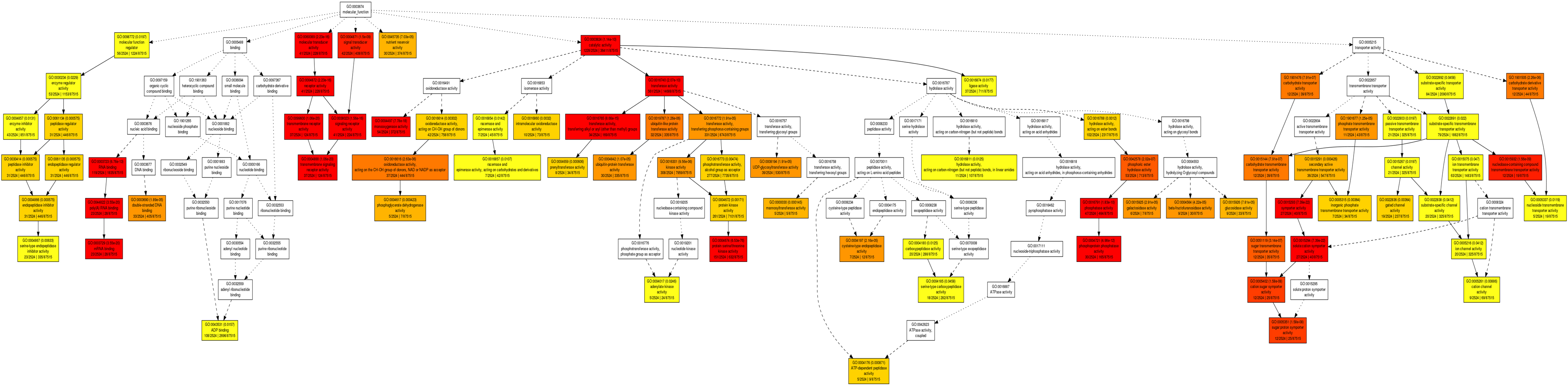


A

B

**Figure S1.** GO enrichment analysis of DEGs in ABD vs A (A. Up-regulated; B. Down-regulated).


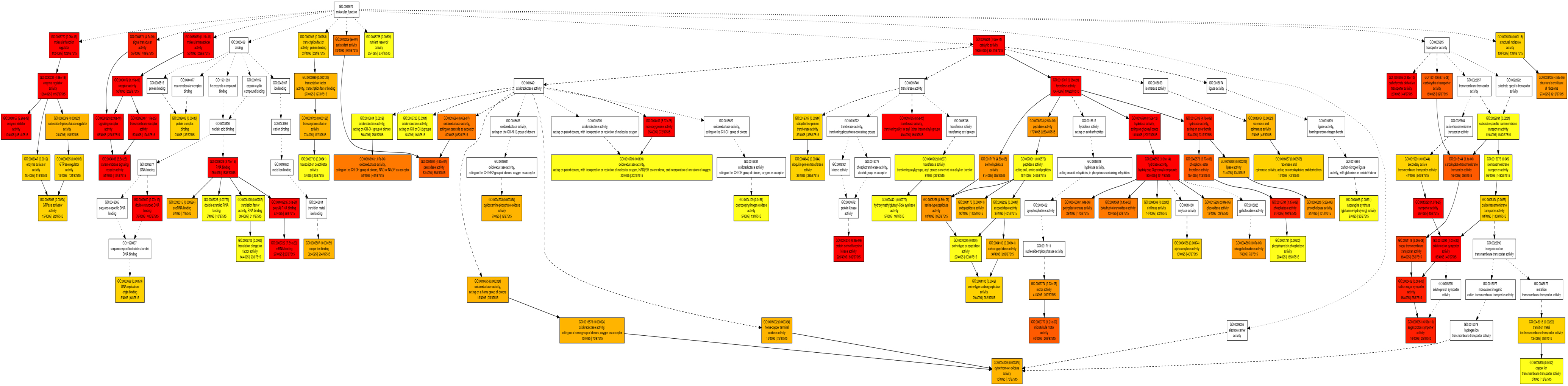

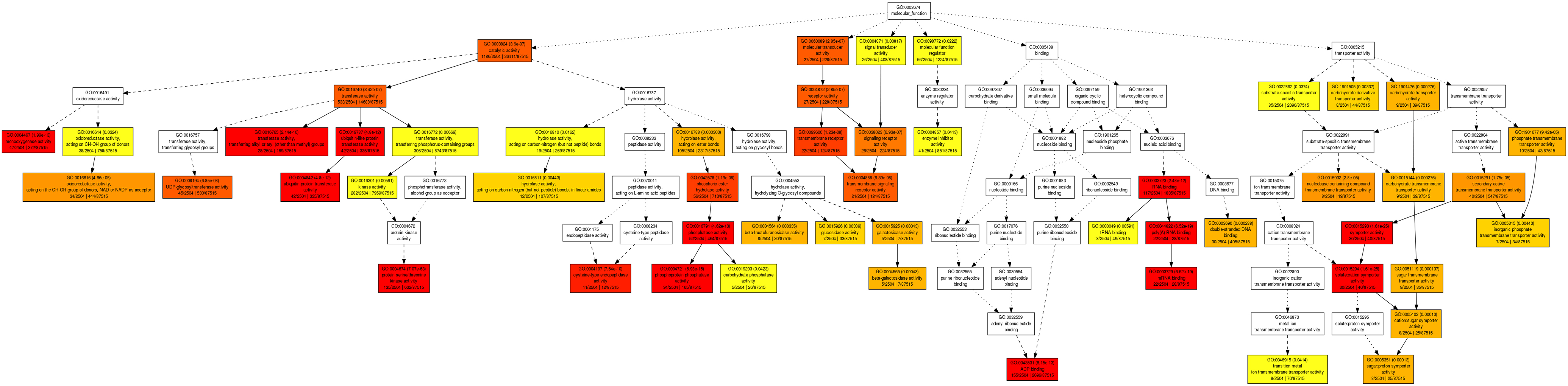


A

B

**Figure S2.** GO enrichment analysis of DEGs in ABD vs B (A. Up-regulated; B. Down-regulated).


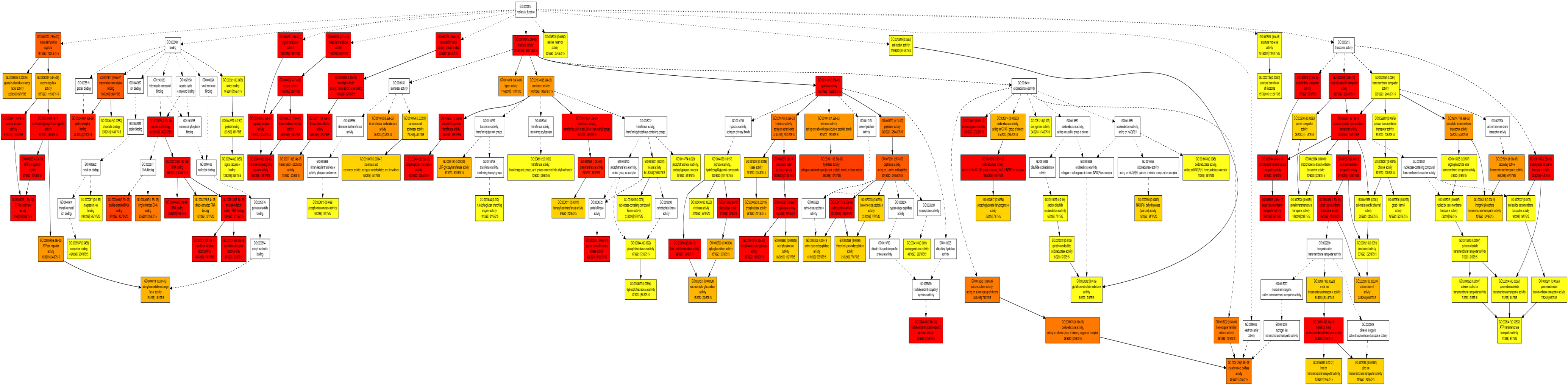

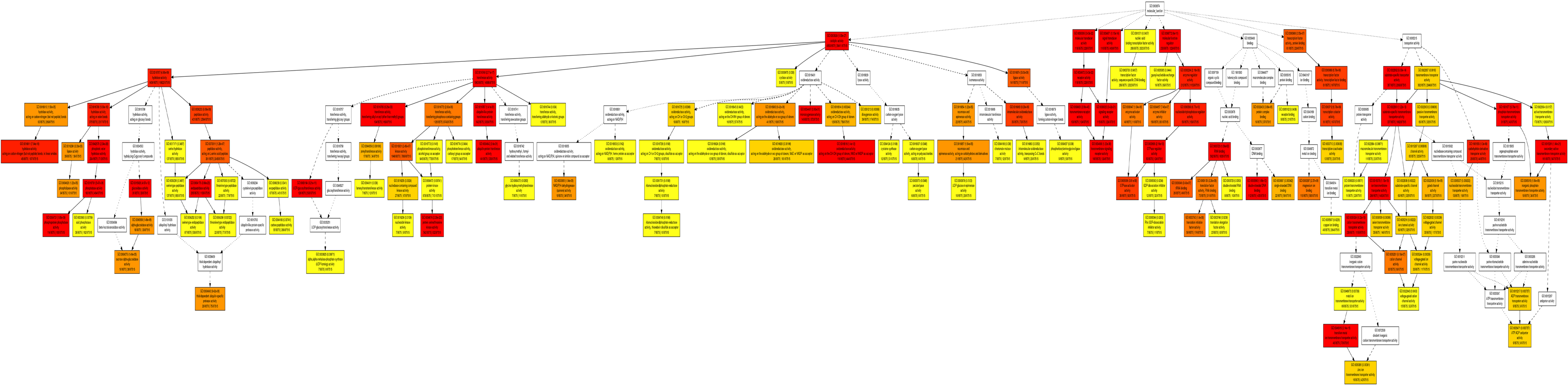


A

B

**Figure S3.** GO enrichment analysis of DEGs in ABD vs B (A. Up-regulated; B. Down-regulated).


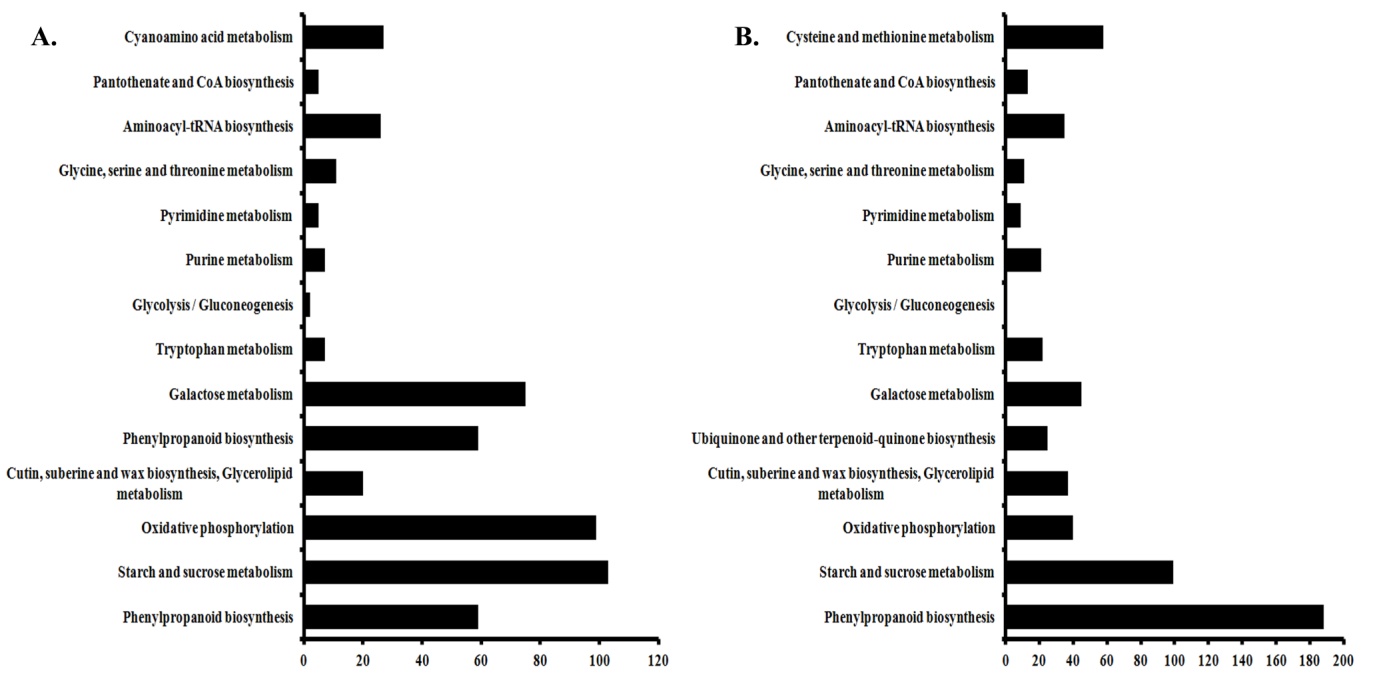


**Figure S4.** Clusters of KEGG functional classification of DEGs in ABD vs A (A. Up regulated B. Down regulated).


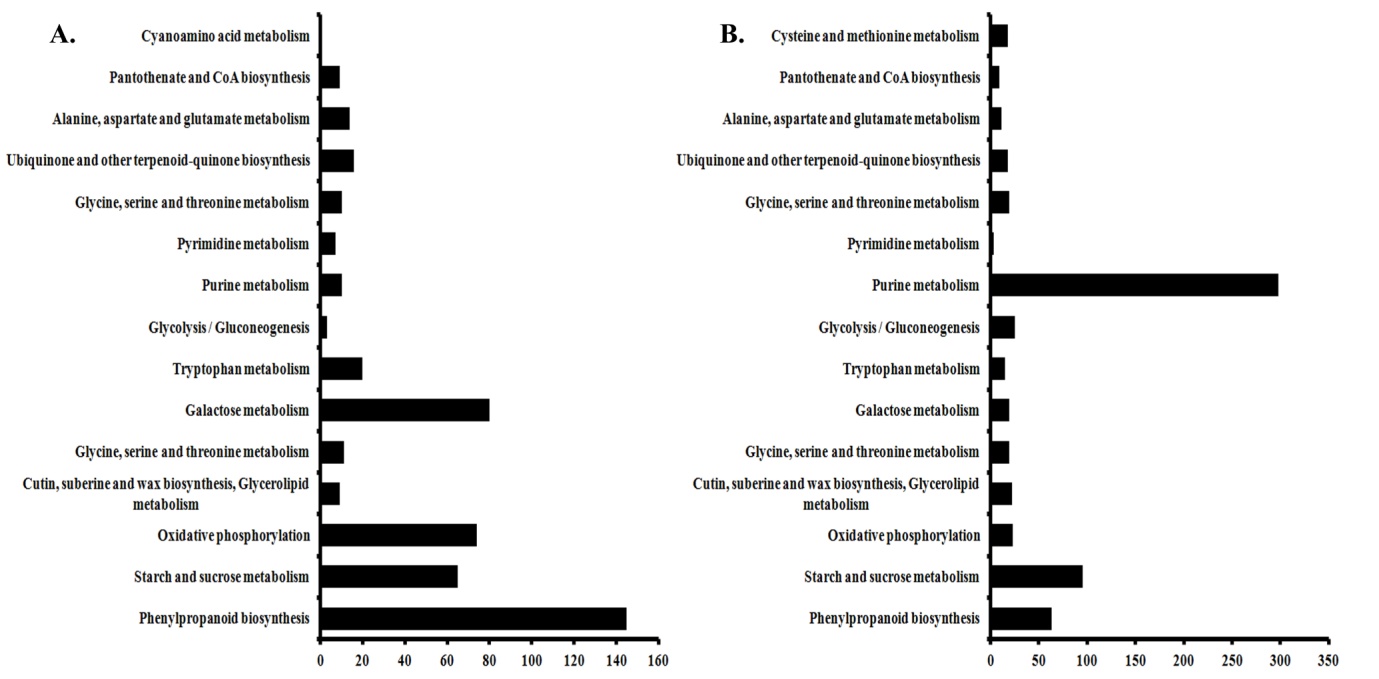


**Figure S5.** Clusters of KEGG functional classification of DEGs in ABD vs B (A. Up regulated B. Down regulated).


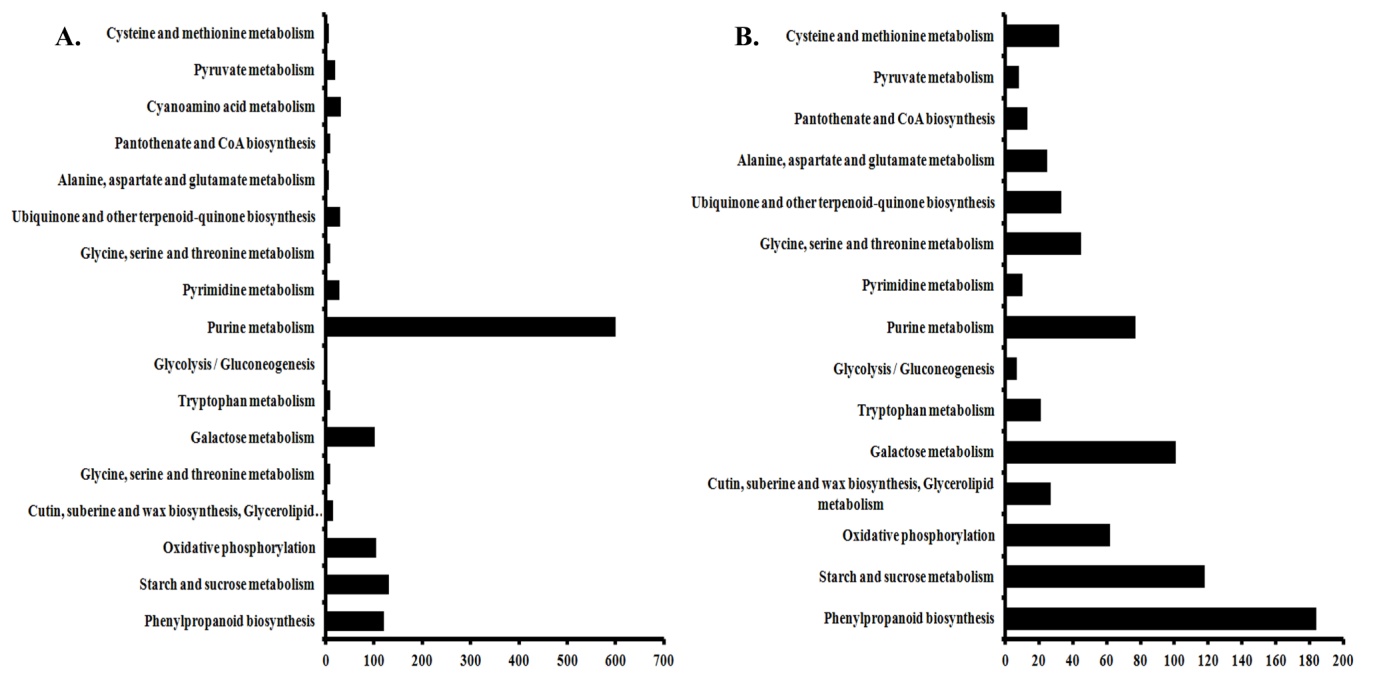


**Figure S6.** Clusters of KEGG functional classification of DEGs in ABD vs D (A. Up regulated B. Down regulated).


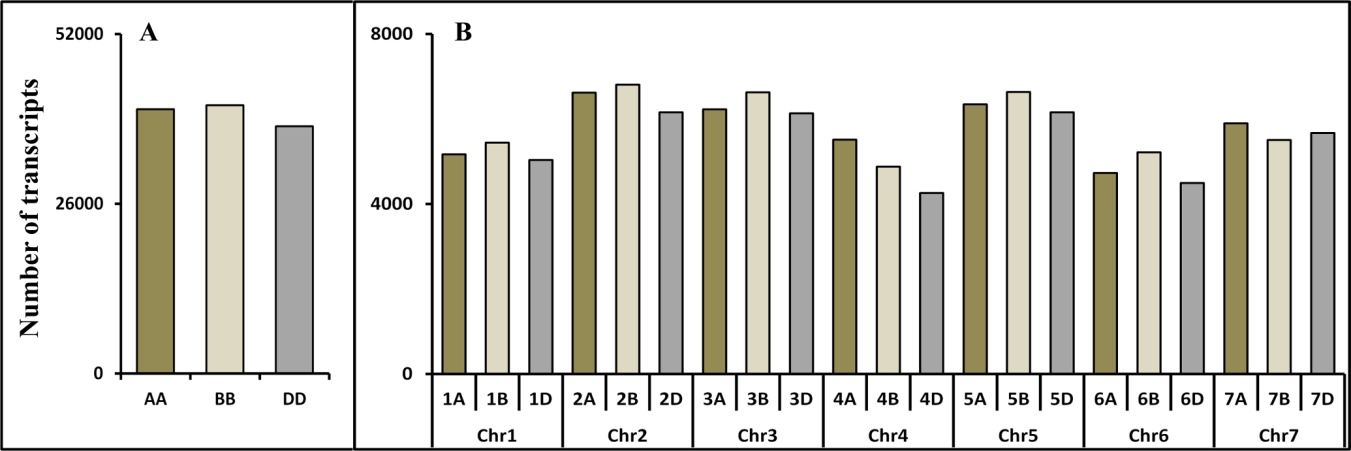


**Figure S7.** Total number of transcripts in subgenomes of hexaploid wheat. B. Number of transcripts distribution in 21 chromosomes.

**Table S1.** List of genes selected for validation and their primer sequences.

| **Gene Name** | **Transcript ID** | **Comparison** | **Primer** |
| --- | --- | --- | --- |
| Avenin a5 | TraesCS7D02G032100 | ABD vs. A | \| F-CTATAGCATTACCATGACG \| \| --- \| \| R- GGCACGAGCTTTATTTAG \| |
| Low molecular weight glutenin | TraesCS1A02G008000 | ABD vs. A | F- GGCAACAACCTCAACAACAA  R- GCACATTGACACTGCACATT |
| Beta-D-xulosidase | TraesCS2A02G523300 | ABD vs. A | F- CATGTGCTCCTACAACAA  R- GATGTATCCGTTGAGCTTCC |
| Beta glucosidases | TraesCS3B02G484000 | ABD vs. A | F-CTATGTGAACGATCGCCCTTTA  R-GGTACTGCAACACACATTGTAATC |
| Alpha humulene synthase | TraesCS2B02G160400 | ABD vs. A | F-CAACTAAATGCGGCCATACAAA  R-GGACATAGGCAACATGGTAAGT |
| Profilin A | TraesCS1B02G356700 | ABD vs. A  ABD vs B | F-CCATCAATATAACGGCGTCCA  R-TTGTTAGGGCAACTGGTCATC |
| Gamma gliadin | TraesCS1D02G008600 | ABD vs. B  ABD vs. D | \| F-TCCAACGACCTCAACAACAA \| \| --- \| \| R-GCAATGGAAGTCATCAACTCAAG \| |
| Avenin | TraesCS4A02G453400 | ABD vs. B  ABD vs. D | F- TCCGATTGAGATAACGAGGA  R- TAGCGGTCATAGGGATGTT |
| Vicilin | TraesCS2B02G389800 | ABD vs. B | \| F- CACTGGCTAATTGGGAAGAA \| \| --- \| \| R- ATCATCCGTTGATGGTAAGC \| |
| Vestitone rductase | TraesCS7D02G493000 | ABD vs. B | F-TGGGCTTCAGGTACAAGTATG  R-AGAGGATACTGTGATCACTTGC |
| Syn-copalyl diphosphate synthase | TraesCS2B02G565800 | ABD vs. B  ABD vs. D | F-GGACATGGAGATGCAAGAACTA  R-AAGACGACCTTTGCGATGT |
| Subtilisn chemotrypsin inhibitor | TraesCS1B02G356700 | ABD vs. D | F-CCATCAATATAACGGCGTCCA  R- TTGTTAGGGCAACTGGTCATC |
| Beta-glucosidase | TraesCS3D02G391900 | ABD vs. D | F- GCTGTCGCACAAGCAATAAA  R- TGAGAGCCCGTTCTTGTAATC |
| Transcription factot MyC2 | TraesCS5A02G306200 | ABD vs. D | F-TCGTGTCTCCATGGAATAAATCAG  R-CAAGCATATAACACTAGCAACACAC |

**Table S2.** List of genes selected for stage specific expression and their primer sequences.

| **Gene Name** | **Category** | **Primer** |
| --- | --- | --- |
| Sulfur-rich seed storage protein | Nutrient reservoir | \| F-AAATGGTGGAAGGGAGGTTG \| \| --- \| \| R-TCACGCTGAAATCCGAAGAT \| |
| Alpha/beta gliadin |  | \| F-AGCCTCAGCAACAATATCCA \| \| --- \| \| R-TGCAGCGCTAGGTTACTTATT \| |
| Germins |  | \| F-TGCTGCGACATTGCTCTT \| \| --- \| \| R-CGTTCTCGTGCACTTTGGA \| |
| Gamma gliadin-B |  | \| F-CAAGCTCCGGTTGAGATCAT \| \| --- \| \| R-ACATTGACTCACAGACCCATC \| |
| High molecular weight glutenin |  | \| F- CCCAAGTGTAACTTCTTC \| \| --- \| \| R- GGAGAAGTTGGGTAGTAT \| |
| Alpha-glucosidase | Carbohydrate metabolism | F-TCCATCAACACCATCCTCAA  R-AATGCGCCAAGCTGAATC |
| Alpha-amylase |  | F-CGAGGGCGATCTCTATGTT  R-TCCCAGACACAGTAGTTGTT |
| Beta- glucosidase |  | F-GGAGCACATTGGATGCATTA  R-TCGAAGTCGACACGATACAT |
| Granule bound starch synthase |  | F-ATCCTCGACCTCAACAACAA  R-CCTATAGATGCCACTGGACTG |
| Beta-amylase |  | \| F- CAGAACTATGCCACTTTC \| \| --- \| \| R- CGGTATTCTTATCAAAGGG \| |
| Trypsin inhibitor CMe | Defence protein | F-ATGTTGAAGAGGCGGTGTT  R-AACTGGGTATGTCCTGGAGTA |
| Thionin |  | F-ACAACAGCCGTCAACTACT  R-ATCATGACATCCACTGGTACA |
| Bowman trypsin inhibitor |  | F-GCAACGACAAGGTGAAGAAG  R-TAGATCTCGGTGCACTTGG |
| Subtilisn chymotrypsin inhibitor |  | F-AAGCCAGCAGTGCTAAGA  R-ATTTAAGTCCTGGGTCACCT |
| Wheat monomeric amylase inhibitor |  | F-GCGAATCTGCATCTTAATTGG  R-TCAACAGGAACAAGTTCACA |
